# Comparison of clinical outcomes between intense pulsed light therapy using two different filters in meibomian gland dysfunction: prospective randomized study

**DOI:** 10.1038/s41598-023-33526-z

**Published:** 2023-04-24

**Authors:** Joon Hyuck Jang, Koeun Lee, Sang Hyu Nam, Jin Kim, Jae Yong Kim, Hungwon Tchah, Hun Lee

**Affiliations:** Department of Ophthalmology, Asan Medical Center, University of Ulsan College of Medicine, 88, Olympic-Ro 43-Gil, Songpa-Gu, Seoul, 05505 South Korea

**Keywords:** Eye diseases, Corneal diseases, Eyelid diseases

## Abstract

Our study compared treatment efficacy between cut-off and notch filters in intense pulsed light (IPL) therapy for meibomian gland dysfunction (MGD) through a prospective, randomized paired-eye trial. Additionally, the efficacy of IPL treatment alone was investigated by restricting other conventional treatments. One eye was randomly selected for an acne filter and the other for a 590-nm filter. Identical four regimens of IPL treatments were administered. The tear break-up time (TBUT), Oxford scale, Sjögren’s International Clinical Collaborative Alliance (SICCA) staining score, tear matrix metalloproteinase-9 (MMP-9) expression, tear osmolarity, and Ocular Surface Disease Index (OSDI) questionnaires were evaluated before and after IPL. Meibomian gland (MG) parameters were measured. When combining the results from both filters, the TBUT, SICCA staining score, OSDI score, and upper and lower lid meibum expressibility were improved after IPL. No significant differences were found between the two filters in the TBUT, Oxford scale, SICCA staining score, MMP-9 expression, tear osmolarity, and MG parameters. Although not significant, the acne filter showed better treatment efficacy than that in the 590-nm filter. IPL alone is efficacious in terms of ocular surface parameters, MG function, and subjective symptoms. Regarding filter selection, both acne and 590-nm filters are promising options for MGD treatment.

## Introduction

Meibomian gland dysfunction (MGD) is a leading cause of dry eye syndrome. The Tear Film and Ocular Surface Society achieved a consensus in 2011 for the definition of MGD as a chronic, diffuse abnormality of meibomian glands, commonly characterized by terminal duct obstruction and/or qualitative/quantitative changes in glandular secretion^1,2^.

The core mechanisms of terminal duct obstruction are hyper-keratinization and altered lipid profile of meibum^3^. Non-polar lipids are decreased, whereas polar lipids, ω-hydroxy fatty acids, and free fatty acids (FFA) are increased; these changes may result from an increased diversity in the bacterial community in patients with MGD^4,5^. FFA, products of lipid break down, are known to stimulate hyper-keratinization^6^. Additionally, changes in lipid profile induce an increase in the melting point and aggravate keratinization by increasing the viscosity of meibum^7^.

Conventional treatments for MGD include warm compresses, lid cleansing^8,9^, antibiotics for lid commensal bacteria^10,11^ or co-existing *Demodex*-related anterior blepharitis*,* anti-inflammatory agents (topical cyclosporine A, topical loteprednol, and oral minocycline)^12–16^, and supplementation with omega-3^17–19^. Procedures for MGD management have also been introduced, such as thermal pulsation (electronic heating device, LipiFlow)^20–22^ and mechanical manipulation of the meibomian gland, such as meibomian gland expression (MGX) and intraductal probing^8,23,24^. Conventional treatments have demonstrated strong therapeutic effects but only in the short-term^25^.

In 2015, intense pulsed light (IPL) was first proposed as a novel, emerging treatment for MGD^26,27^. The mechanism by which IPL acts as an MGD treatment remains unclear; however, the primary speculative theories suggest abnormal blood vessel thrombosis, meibum liquefaction, photo-modulation, and *Demodex* eradication^27–30^. During IPL treatment, physicians can control the penetration depth and selective chromophore targeting through the selection of specific filters. Filters are generally classified into two groups: cut-off and notch. Cut-off filters, such as the 590-nm filter, block wavelengths under a specified number. In contrast, acne filters are a representative type of notch filter that induce the IPL to emit wavelengths of 400–600 nm and 800–1200 nm by blocking 600–800 nm^31^. Although we previously retrospectively demonstrated the efficacy and safety of IPL with an acne filter^32^, there is currently no prospective, randomized clinical trial comparing the clinical outcomes between 590-nm and acne filters for the treatment of moderate to severe MGD. Therefore, this study aimed to compare the treatment efficacy of 590-nm and notch filters through a prospective, randomized paired-eye trial. Additionally, we investigated the effects of IPL alone by restricting access to other treatments.

## Results

### Ocular surface parameters

This study included 30 consecutive patients (60 eyes), six male (12 eyes) and 24 female (48 eyes) individuals, with a mean age of 66.0 ± 8.8 years (range 43–80). Four weeks after the last IPL treatment during which other conventional MGD treatments were restricted, the tear break-up time (TBUT) (from 2.09 ± 1.16 to 4.08 ± 1.52, p < 0.001), Sjögren's International Collaborative Clinical Alliance (SICCA) staining score (from 6.50 ± 2.16 to 3.00 ± 1.87, p < 0.001), Oxford staining score (from 2.65 ± 1.05 to 0.93 ± 0.73, p < 0.001), and Ocular Surface Disease Index (OSDI) score (from 61.69 ± 20.62 to 40.48 ± 19.07, p < 0.001) significantly improved when combining the results from both filters (Table 1). However, tear production was not improved after IPL treatment. In each filter setting, the TBUT, SICCA staining score, and Oxford staining score significantly improved (Table 2). No significant difference was found between the two filters in ocular surface parameters (Table 3). Although not statistically significant, the improvements in TBUT (1.91 ± 1.40 in the 590-nm filter and 2.08 ± 1.61 in the acne filter), SICCA staining score (− 3.20 ± 2.34 in the 590-nm filter and − 3.80 ± 2.77 in the acne filter), and Oxford staining score (− 1.70 ± 1.02 in the 590-nm filter and − 1.73 ± 1.17 in the acne filter) were higher in the IPL with acne filter group than those in the IPL with 590-nm filter group (Table 3).

**Table 1:** Comparison of clinical signs and symptoms before treatment and 4 weeks after the last treatment of intense pulsed light (IPL) regardless of filter type (590-nm or acne filter) in patients with moderate to severe meibomian gland dysfunction.

Parameters		Before treatment (n = 60 eyes)	4 weeks after last IPL treatment (n = 60 eyes)	*p* value
TBUT (sec)		2.09 (1.16)	4.08 (1.52)	< 0.001
SICCA staining score		6.50 (2.16)	3.00 (1.87)	< 0.001
Oxford staining score		2.65 (1.05)	0.93 (0.73)	< 0.001
Schirmer’s test		7.97 (5.10)	8.02 (5.34)	0.943
OSDI score		61.69 (20.62)	40.48 (19.07)	< 0.001
Lid margin telangiectasia	Upper eyelid (0–3)	2.28 (0.69)	0.95 (0.75)	< 0.001
	Lower eyelid (0–3)	2.28 (0.69)	0.96 (0.69)	< 0.001
Meibum expressibility	Upper eyelid (0–3)	3.78 (2.26)	7.31 (1.49)	< 0.001
	Lower eyelid (0–3)	3.80 (2.23)	7.33 (1.31)	< 0.001
Meibum secretion score	Upper eyelid (0–3)	19.23 (4.13)	8.87 (5.29)	< 0.001
	Lower eyelid (0–3)	19.62 (3.09)	9.92 (5.10)	< 0.001
Lid wiper epitheliopathy staining grade	Upper eyelid (0–3)	2.28 (0.59)	1.54 (0.48)	< 0.001
Tear osmolarity		308.95 (16.30)	306.00 (22.40)	0.367
Tear MMP-9 level		2.95 (1.14)	2.43 (0.96)	0.002
Tear MMP-9 positivity		61.7% (37/60)	41.7% (25/60)	0.017

Results are presented as mean (standard deviation).

IPL, intense pulsed light; TBUT, tear film break-up time; SICCA, Sjögren's International Clinical Collaborative Alliance; OSDI, ocular surface disease index; MMP-9, matrix metalloproteinase-9.

**Table 2:** Comparison of clinical signs and symptoms before treatment and 4 weeks after the last treatment of intense pulsed light (IPL) with each filter (590-nm or acne filter) in patients with moderate to severe meibomian gland dysfunction.

Parameters		590-nm filter (n = 30 eyes)			Acne filter (n = 30 eyes)		
		Before treatment	4 weeks after last IPL treatment	*p* value	Before treatment	4 weeks after last IPL treatment	*p* value
TBUT (sec)		2.16 (1.20)	4.07 (1.48)	< 0.001	2.02 (1.13)	4.10 (1.58)	< 0.001
SICCA staining score		6.37 (2.08)	3.17 (1.78)	< 0.001	6.63 (2.27)	2.83 (1.97)	< 0.001
Oxford staining score		2.67 (0.99)	0.97 (0.72)	< 0.001	2.63 (1.13)	0.90 (0.76)	< 0.001
Schirmer’s test		8.27 (5.63)	8.37 (5.95)	0.929	7.67 (4.57)	7.67 (4.74)	> 0.999
Lid margin telangiectasia	Upper eyelid (0–3)	2.27 (0.69)	0.97 (0.76)	< 0.001	2.30 (0.70)	0.93 (0.74)	< 0.001
	Lower eyelid (0–3)	2.27 (0.69)	0.97 (0.67)	< 0.001	2.30 (0.70)	0.97 (0.72)	< 0.001
Meibum expressibility	Upper eyelid (0–3)	3.87 (2.21)	7.27 (1.68)	< 0.001	3.70 (2.35)	7.37 (1.30)	< 0.001
	Lower eyelid (0–3)	3.83 (2.28)	7.30 (1.32)	< 0.001	3.77 (2.22)	7.37 (1.33)	< 0.001
Meibum secretion score	Upper eyelid (0–3)	19.53 (4.08)	9.27 (5.53)	< 0.001	18.93 (4.23)	8.47 (5.10)	< 0.001
	Lower eyelid (0–3)	19.70 (3.12)	10.57 (5.20)	< 0.001	19.53 (3.10)	9.27 (5.00)	< 0.001
Lid wiper epitheliopathy staining grade	Upper eyelid (0–3)	2.27 (0.61)	1.52 (0.48)	< 0.001	2.28 (0.58)	1.57 (0.49)	< 0.001
Tear osmolarity		307.93 (17.62)	305.37 (15.29)	0.503	309.97 (15.10)	306.63 (28.04)	0.537
Tear MMP-9 level		2.90 (1.16)	2.40 (0.93)	0.033	3.00 (1.14)	2.47 (1.01)	0.033
Tear MMP-9 positivity		60.0% (18/30)	43.3% (13/30)	0.134	63.3% (19/30)	40.0% (12/30)	0.032

Results are presented as mean (standard deviation).

IPL, intense pulsed light; TBUT, tear film break-up time; SICCA, Sjögren's International Clinical Collaborative Alliance; MMP-9, matrix metalloproteinase-9.

**Table 3:** Comparison of clinical parameters after intense pulsed light (IPL) treatment between acne and 590-nm filters.

Parameters	Filter	Mean difference (post minus pre-treatment)	*p* value
TBUT (sec)	590-nm filter	1.91 (1.41)	0.652
	Acne filter	2.08 (1.61)	
SICCA staining score	590-nm filter	− 3.20 (2.34)	0.369
	Acne filter	− 3.80 (2.77)	
Oxford staining score	590-nm filter	− 1.70 (1.02)	0.907
	Acne filter	− 1.73 (1.17)	
Schirmer test	590-nm filter	0.10 (6.13)	0.943
	Acne filter	0.00 (4.65)	
Upper lid margin telangiectasia	590-nm filter	− 1.30 (0.99)	0.780
	Acne filter	− 1.37 (0.85)	
Lower lid margin telangiectasia	590-nm filter	− 1.30 (0.92)	0.875
	Acne filter	− 1.33 (0.71)	
Upper lid meibum expressibility	590-nm filter	3.40 (2.61)	0.676
	Acne filter	3.67 (2.29)	
Lower lid meibum expressibility	590-nm filter	3.47 (2.27)	0.811
	Acne filter	3.60 (2.01)	
Upper lid meibum secretion score	590-nm filter	− 10.27 (7.07)	0.910
	Acne filter	− 10.47 (6.55)	
Lower lid meibum secretion score	590-nm filter	− 9.13 (5.88)	0.448
	Acne filter	− 10.27 (5.61)	
Lid wiper epitheliopathy staining grade	590-nm filter	− 0.75 (0.76)	0.862
	Acne filter	− 0.72 (0.72)	
Tear osmolarity	590-nm filter	− 2.57 (20.74)	0.907
	Acne filter	− 3.33 (29.20)	
Tear MMP-9 level	590-nm filter	− 0.50 (1.22)	0.919
	Acne filter	− 0.53 (1.31)	

Results are presented as mean (standard deviation).

TBUT, tear film break-up time; SICCA, Sjögren's International Clinical Collaborative Alliance; MMP-9, matrix metalloproteinase-9.

### Meibomian gland (MG) parameters

Four weeks after the last IPL treatment, when combining the results from both filters, upper lid margin telangiectasia (from 2.28 ± 0.69 to 0.95 ± 0.75, p < 0.001) and lower lid margin telangiectasia (from 2.28 ± 0.69 to 0.96 ± 0.69, p < 0.001) improved (Table 1). Additionally, upper lid meibum expressibility (from 3.78 ± 2.26 to 7.31 ± 1.49, p < 0.001) and lower lid meibum expressibility (from 3.80 ± 2.23 to 7.33 ± 1.31, p < 0.001) improved, explaining the improvement in MG orifice obstruction. The upper lid meibum secretion score (from 19.23 ± 4.13 to 8.87 ± 5.29, p < 0.001) and lower lid meibum secretion score (from 19.62 ± 3.09 to 9.92 ± 5.10, p < 0.001) were improved, explaining the improvement in meibum quality. Lid wiper epitheliopathy (LWE) staining grade (from 2.28 ± 0.59 to 1.54 ± 0.48, p < 0.001) also improved (Table 1). In each filter setting, all MG parameters significantly improved (Table 2). No significant differences were found between the two filters in MG parameters. Although the difference was not statistically significant, compared with those in the IPL with 590-nm filter group, the IPL with acne filter group showed larger improvements in lid margin telangiectasia, lid meibum expressibility, and lid meibum secretion score (Table 3).

### Tear osmolarity and tear MMP-9 levels

When combining the results from both filters, tear osmolarity showed no significant improvement after IPL treatment. In contrast, tear MMP-9 levels significantly improved (from 2.95 ± 1.14 to 2.43 ± 0.96, *p* = 0.002; Table 1). Tear MMP-9 positivity also decreased from 61.7% (37/60) to 41.7% (25/60) (*p* = 0.017). In each filter setting, the tear MMP-level significantly improved (Table 2). Tear MMP-9 positivity significantly decreased in the acne filter group alone (*p* = 0.032).

### Clinical characteristics of IPL responding eyes

According to the subgroup analysis based on the improvement in MG expressibility, the average age was younger in the 590-nm filter IPL-responding subgroup than that in the non-responding subgroup (62.35 ± 7.92 years, responding subgroup; 70.69 ± 7.90 years, non-responding subgroup; *p* = 0.001). The TBUT was significantly lower in the acne filter responding subgroup than that in the non-responding subgroup (*p* = 0.020), indicating that the ocular surface was more unstable in the acne filter IPL-responding subgroup than that in the non-responding subgroup. Upper lid expressibility was significantly lower in the acne filter responding subgroup than that in the non-responding subgroup (*p* = 0.018), indicating that MG orifice obstruction for the upper eyelid was more severe in the acne filter IPL-responding subgroup than that in the non-responding subgroup. (Table 4). Lower lid expressibility was significantly lower in the responding subgroups than that in the non-responding subgroups in both filter groups (all p < 0.001), indicating that MG orifice obstruction for the lower eyelid was more severe in the responding group than that in the non-responding group (Table 4). The lower lid meibum secretion score was significantly higher in the responding subgroups than that in the non-responding subgroups in both filter groups (*p* = 0.032 for the 590-nm filter and *p* = 0.003 for the acne filter), indicating that the meibum quality for the lower eyelid had a more toothpaste-like consistency in the responding subgroup than in the non-responding subgroup. However, no ocular or dermatologic adverse events were observed in any patient.

**Table 4:** Comparison of clinical parameters between acne and 590-nm filters in the responding and non-responding groups.

Parameters	Filter	Responding group	Non-responding group	*p* value
	590-nm, n (%)	17 (56.7%)	13 (43.3%)	> 0.999
	Acne, n (%)	17 (56.7%)	13 (43.3%)	
Sex	590-nm	4/13 (M/F)	2/11 (M/F)	0.672
	Acne	5/12 (M/F)	1/12 (M/F)	0.196
Age	590-nm	62.35 (7.92)	70.69 (7.90)	0.001
	Acne	65.82 (8.57)	66.15 (9.52)	0.805
TBUT (sec)	590-nm	1.89 (1.16)	2.52 (1.19)	0.182
	Acne	1.65 (1.00)	2.50 (1.14)	0.020
SICCA staining score	590-nm	6.59 (1.94)	6.08 (2.29)	0.526
	Acne	6.94 (2.14)	6.23 (2.45)	0.538
Oxford staining score	590-nm	2.53 (0.94)	2.85 (1.07)	0.343
	Acne	2.59 (0.94)	2.69 (1.38)	0.745
Schirmer test	590-nm	8.77 (5.90)	7.62 (5.42)	0.364
	Acne	8.18 (4.20)	7.00 (5.12)	0.129
Upper lid margin telangiectasia	590-nm	2.41 (0.62)	2.08 (0.76)	0.216
	Acne	2.41 (0.62)	2.15 (0.80)	0.384
Lower lid margin telangiectasia	590-nm	3.18 (1.85)	4.77 (2.39)	0.216
	Acne	2.47 (0.62)	2.08 (0.76)	0.143
Upper lid Meibum expressibility	590-nm	3.18 (1.85)	4.77 (2.39)	0.051
	Acne	2.82 (2.30)	4.85 (1.95)	0.018
Lower lid meibum expressibility	590-nm	2.41 (1.18)	5.69 (2.02)	< 0.001
	Acne	2.35 (1.41)	5.62 (1.66)	< 0.001
Upper lid meibum secretion score	590-nm	20.12 (4.26)	18.77 (3.88)	0.252
	Acne	19.41 (4.37)	18.31 (4.13)	0.320
Lower lid meibum secretion score	590-nm	20.88 (1.87)	18.15 (3.78)	0.032
	Acne	21.00 (2.09)	17.62 (3.23)	0.003
Lid wiper epitheliopathy staining grade	590-nm	2.41 (0.59)	2.08 (0.61)	0.159
	Acne	2.41 (0.59)	2.12 (0.55)	0.172
Tear Osmolarity	590-nm	306.18 (15.06)	310.23 (20.93)	0.516
	Acne	309.71 (14.00)	310.31 (17.02)	0.785
Tear MMP-9 level	590-nm	2.71 (1.21)	3.15 (1.07)	0.331
	Acne	2.94 (1.30)	3.08 (0.95)	0.680

Results are presented as mean (standard deviation).

IPL, intense pulsed light; TBUT, tear film break-up time; SICCA, Sjögren's International Clinical Collaborative Alliance; MMP-9, matrix metalloproteinase-9.

## Discussion

The current study showed that even without anti-inflammatory eye drops or eyelid management, IPL treatment significantly improved clinical symptoms and signs of MGD, including the OSDI score, TBUT, ocular staining scores, and MG parameters. Although the acne filter showed better treatment efficacy than the 590-nm filter, there were no significant differences in the TBUT, Oxford scale, SICCA staining score, MMP-9 expression, tear osmolarity, and MG parameters between both filters. Additionally, regardless of filter type, the responding subgroup showed worse baseline lower lid meibum expressibility and lower lid meibum secretion scores than those in the non-responding subgroup. Interestingly, in the acne filter group, the responding subgroup showed worse baseline TBUT and upper lid meibum expressibility than those in the non-responding subgroup.

Several meta-analyses reported that the TBUT and Standard Patient Evaluation of Eye Dryness (SPEED) scores improved after IPL treatment in patients with MGD. However, the SPEED score improvement was not significant^33–35^. As shown in one meta-analysis, the therapeutic effect of IPL on subjective symptom scores is controversial^33^. We assumed that many confounding factors such as warm compresses, lid scrubs, and topical anti-inflammatory eyedrops could contribute to ambiguous symptomatology. Most previous studies did not control for patient’s self-administration of eye drops, which could be an important confounding factor^36^. Similarly, recent studies that controlled for the self-administration of eye drops did not control for self-administrated eyelid management, such as warm compresses and lid scrubs^37^. Therefore, at every visit from the start of IPL treatment in the current study, we instructed patients to apply artificial tears only and to not use warm compresses. Thus, the strength of our study comes from a precise evaluation of the therapeutic effect of IPL alone, through the strict control of self-administration of eye drops and eyelid management.

The mechanism underlying IPL treatment remains unclear. In general, the IPL is considered to liquify meibum through heating. Other suggested mechanisms for IPL include photo-modulation, eradication of *Demodex,* and thrombosis of vessels. Photo-modulation is the ability of IPL to activate fibroblasts and enhance collagen synthesis through stimulating biologic patterns via different wavelength light sources^38^. The most important mechanism regulating IPL is the thrombosis of superficial vessels^39^. Throughout vascular thrombosis, IPL can destruct lid telangiectasia, which is a key feature of MGD. In particular, with notch filters including acne and vascular filters, filtering wavelengths between 670 and 870 nm improve the ratio of vascular-to-pigment destruction^40^. We recently demonstrated that IPL treatment with acne or vascular filter showed strong therapeutic efficacy with minimal adverse events^32,41^. Similarly, our current study showed that IPL with an acne filter had an improved therapeutic effect on the TBUT, ocular staining scores, lid margin telangiectasia, lid meibum expressibility, lid meibum secretion score, LWE staining grade, and tear MMP-9 level.

Few studies have compared IPL filters for MGD treatment, and no studies have considered direct comparisons between the filters. To our knowledge, this study is the first to directly compare two filters in a prospective, randomized, paired-eye study. IPL treatment with both 590-nm and acne filters showed good therapeutic efficacy for MGD; however, no significant differences were observed in the clinical parameters between the 590-nm and acne filters. As previously mentioned, IPL with an acne filter emits 400–600 nm and 800–1200 nm of wavelength energy, unlike a conventional cut-off filter (590-nm filter). The light between the two bands (600–800 nm) is highly absorbed by melanin and is associated with a risk of epidermal damage and post-inflammatory hyperpigmentation. A dual-band filter enables more selective targeting. For example, in dermatology, the acne filter is applied to acne treatment. The 400–600-nm wavelength energy can target porphyrin and destroy *Cutibacterium acnes*. In addition, the 800–1200-nm wavelength can exert an anti-inflammatory effect and destroy sebaceous glands^31,42^. Therefore, we assumed that similar mechanisms could be applied to MG. In our study, although the differences were not statistically significant, the IPL with acne filter group had larger improvements in lid margin telangiectasia, lid meibum expressibility, and lid meibum secretion score than those in the IPL with 590-nm filter group. Moreover, in the acne filter group alone, the responding subgroup showed worse baseline TBUT and upper lid meibum expressibility than those in the non-responding subgroup.

Although we expected improved efficacy in lid margin telangiectasia via deeper penetration and better vascular-to-pigment destruction ratio with the acne filter, no significant difference was found in lid margin telangiectasia between the two filters. To date, no effective method of evaluating superficial and deep lid margin telangiectasia has been developed. Recent studies, including our study, have depended on a slit lamp examination to evaluate and grade lid telangiectasia, which is limited to examination of the superficial layer. Therefore, to evaluate the effect of IPL with a notch filter (acne or vascular filter) on telangiectasia at a deeper layer, more advanced technology is necessary.

Previous studies on the prognostic factors of IPL have reported that patients with MGD with longer baseline TBUT and less atrophic change in MG showed better therapeutic efficacy^43^. In our study, regardless of filter type, the responding subgroup showed worse baseline lower lid meibum expressibility and lower lid meibum secretion scores than those of the non-responding subgroup, suggesting that obstructive MGD frequently occurred in the responding subgroup. Therefore, we postulate that patients with obstructive MGD showing poor expressibility and secretion scores in the lower lid are good candidates for IPL treatment, regardless of filter type. Interestingly, in the acne filter group, the baseline TBUT and upper lid meibum expressibility were worse in the responding subgroup than those in the non-responding subgroup. Additionally, in contrast to the 590-nm filter group, no difference was observed in age between the responding and non-responding subgroups in the acne filter group. Hence, we can assume that older adult patients with obstructive MGD showing poor expressibility and secretion scores in the lower lid, lower TBUT, and poor expressibility in the upper lid are good candidates for IPL treatment with the acne filter.

This study has some limitations. First, the number of enrolled patients was relatively small. Second, the follow-up period was relatively short-term. Nevertheless, our study may be the first prospective, randomized design to compare the efficacy of IPL treatments between cut-off and notch filters through paired-eye design. Our results also showed a significant therapeutic effect of IPL on MGD under strictly controlled self-administration of other treatment modalities. According to one study, 14% of patients had adverse effects after IPL, including conjunctival cysts, floaters, hair loss, blistering, light sensitivity, and redness^27^. In the current study, no regional or systemic adverse effects occurred in any patient.

In summary, IPL alone is a clinically efficacious treatment for moderate to severe MGD. Regarding the filter selection, both acne and 590-nm filters are promising options for MGD treatment.

## Methods

This prospective, randomized paired-eye study was conducted with the approval of the Institutional Review Board of the Asan Medical Center and the University of Ulsan College of Medicine, Seoul, South Korea (2021–0815). The study adhered to the tenets of the Declaration of Helsinki and followed good clinical practice guidelines. Signed informed consent was obtained from all participants before enrollment. The inclusion criteria were as follows: healthy adult patients aged between 19 to 85 years with moderate-to-severe MGD and a moderate OSDI score of > 23. Diagnosis of moderate to severe MGD was confirmed when patients exhibited moderate to severe symptoms, including ocular discomfort, itching, or photophobia at least 3 months before enrollment and clinical signs of moderate to severe degree MGD. One or more of the following three clinical signs in moderate to severe MGD were necessary: decreased meibum secretion with MG expressor or cloudy/toothpaste-like secretion with MG expressor; > 2 definite telangiectasias in the lid margin; and > 2 obstructions of the MG orifice^1,44^. The exclusion criteria were as follows: Sjögren's syndrome, history of previous intraocular or ocular surgery, glaucoma and receiving topical medication, eyelid malposition, ocular infection, non-dry eye ocular inflammation, ocular allergy, autoimmune disease, use of contact lenses during the study period, receiving clinical skin treatments within 2 months before the study, semi-permanent makeup or tattoos, pigmented lesion at the treatment site, and pregnancy or lactation.

All patients underwent four sessions of IPL (M22; Lumenis, Yokneam, Israel) with meibomian gland expression (MGX) performed by one trained physician (HL) with a 2-week interval between each IPL session. Before the treatment sessions, every patient underwent Fitzpatrick skin typing, and adjustment of the IPL system was performed for a specific setting. All patients enrolled had Fitzpatrick skin type 3^32,41,45^. Clinical parameters for MGD signs were recorded via slit lamp examination before IPL treatment and 4 weeks after four sessions of IPL treatment. Included parameters were the TBUT, SICCA and Oxford staining score for the cornea and conjunctiva, LWE grade, and MG parameters, including meibum expressibility, quality of secretion, and lid margin telangiectasia. Lid margin telangiectasia (0 = no or slight redness in lid margin conjunctiva and no telangiectasia crossing MG orifices; 1 = redness in lid margin conjunctiva and no telangiectasia crossing MG orifices; 2 = redness in lid margin conjunctiva and telangiectasia crossing MG orifices with a distribution of less than half of full length of lid; and 3 redness in lid margin conjunctiva and telangiectasia crossing MG orifices with a distribution of half or more of full length of lid) was assessed and scored for both upper and lower lids^46^. Meibum expressibility was scored from the upper and lower central eight glands as follows: from 8, referring to all eight glands, to 0, referring to zero glands. The quality of the expressed meibum was scored from 0 to 3: 0 = clear fluid; 1 = cloudy fluid; 2 = cloudy fluid with particles; and 3 = opaque, toothpaste-like meibum or solid obstruction with MG plugging. The secretion score was calculated based on expressibility and meibum quality from eight glands in the central eyelids in each upper and lower lid^47^. These scores were summed across the eight glands to obtain a secretion score ranging from 0 to 24. The LWE with upper lid margin fluorescein staining was graded zero to 3 for each of two characteristics; the linear area of staining (0 < 2 mm; 1 = 2–4 mm; 2 = 5–9 mm; and 3 =  ≥ 10 mm) and severity of the staining (0 = absent; 1 = mild; 2 = moderate; and 3 = severe). The total grade for the fluorescein staining of the lid wiper was the average of the grades for the linear area and severity of the staining^48^.

In addition, tear osmolarity, Schirmer’s test I without topical anesthesia, and tear matrix metalloproteinase (MMP)-9 expression were measured. MMP-9 level measurement was performed using the InflammaDry immunoassay (Rapid Pathogen Screening, Inc., Sarasota, FL). One blue line in the control zone with one red line in the result zone indicated a positive test result, whereas one blue line without a red line indicated a negative test result. A red line in the result zone correlated to a concentration of MMP-9 (strong positive, positive, or weak positive). Throughout the grading scale, we could apply semi-quantitative interpretations regarding ocular surface inflammation severity. Because of proportional increases in MMP-9 concentration with signal strength, a more vivid color in the red zone indicates higher MMP-9 levels (0 = none; 1 = trace; 2 = weak positive; 3 = positive; and 4 = strong positive)^49^. When determining the positivity of MMP-9, positive MMP-9 (MMP-9 ≥ 40 ng/mL) was defined as including weak positive, positive, and strong positive, and negative MMP-9 (MMP-9 < 40 ng/mL) was defined as including trace positive and negative^45^. All patients completed an OSDI before and 4-weeks after IPL sessions to report clinical symptoms.

MG expressibility is a key baseline characteristic that correlates with ocular surface parameters and cytokines^50^. We classified patients into two subgroups according to MG expressibility changes in the lower lid after IPL treatment: responding and non-responding subgroups. If the lower lid MG expressibility increased ≥ 4 grades after IPL treatment compared with the grade before IPL treatment, patients were categorized as the responding subgroup. Possible adverse events such as loss of eyelashes, trichiasis, and damage of the iris tissue were evaluated via slit-lamp examination at every visit. Any dermatologic adverse events were evaluated and recorded, such as redness, hyper- or hypopigmentation, blistering, or swelling.

### Statistical analyses

Statistical analysis was performed using SPSS software version 25.0 (IBM, Armonk, NY, USA). The normality of the data distribution was analyzed using the Shapiro–Wilk test. To evaluate clinical efficacy of IPL before and after treatment, paired t-test and McNemar's test were performed. Independent t- and chi-squared tests were performed to compare differences in outcomes between the two filters. Mann–Whitney U and Fisher exact tests were used to compare baseline clinical parameters between the responding and non-responding subgroups. A p value of < 0.05 was considered statistically significant.

### Sample size calculation, randomization, and masking

**Figure 1:**
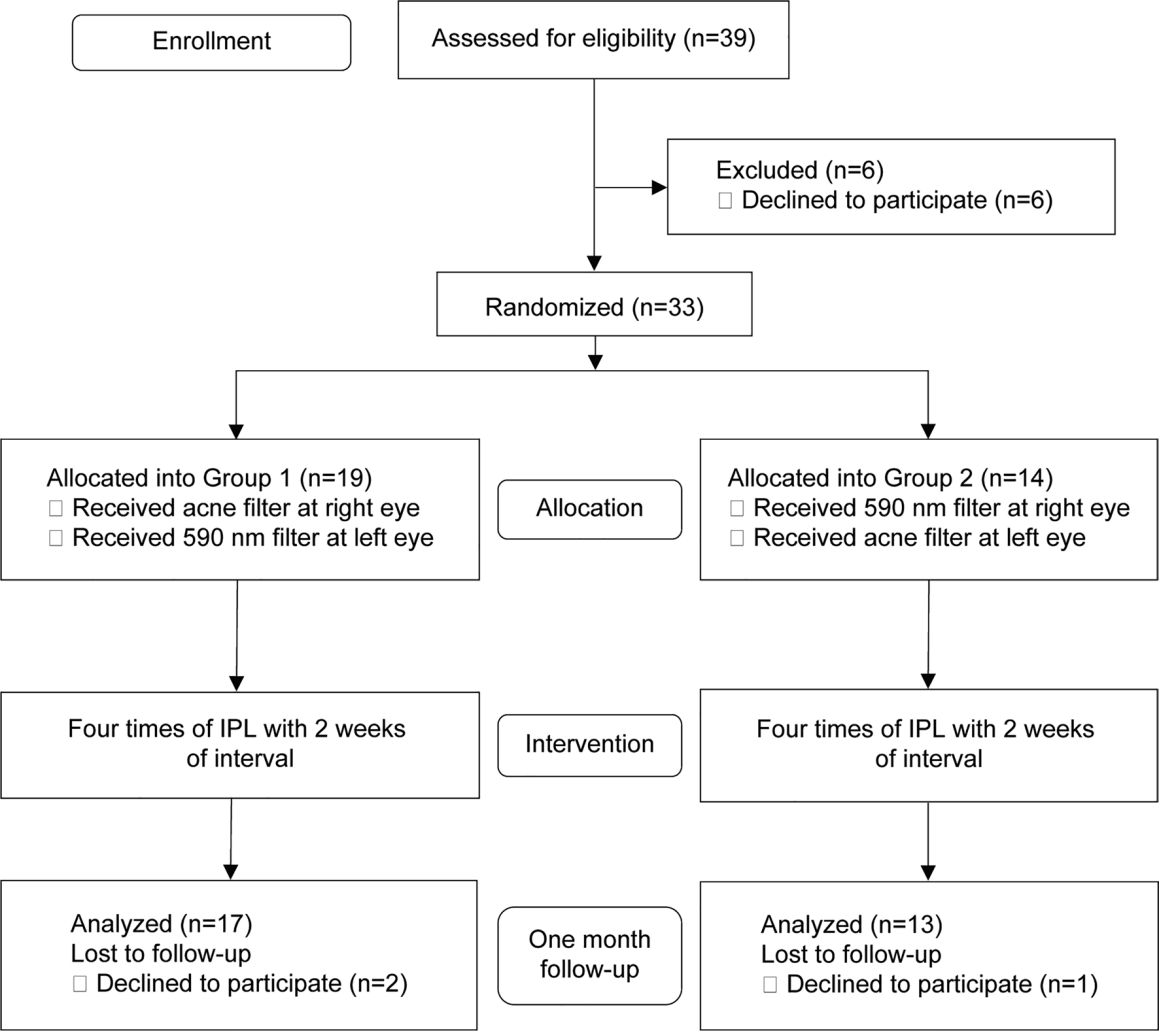
Flow diagram according to the consolidated standards of reporting trials (CONSORT) statement, showing enrollment, randomization, and patient flow in this study for moderate and severe meibomian gland dysfunction.

Sample size was calculated on the basis of assumed mean differences in the MMP-9 grades between the 590-nm and acne filter groups at 4 weeks after the final treatment. With these assumptions, a sample size of 30 eyes per group would yield a power of 90% to show a significant difference with a two-sample t-test. We chose an α level of 0.025 to ensure an overall type I error rate of 0.05 according to the Bonferroni procedure. Considering the drop-out ratio, we opted to recruit 33 patients. A randomization sequence was created using random block sizes of 2, 4, and 6, by an independent doctor. The allocation sequence was concealed from the physician enrolling and assessing patients in sequentially numbered, opaque, and sealed envelopes. Outcome assessors and data analysts were blinded to the allocations. In group I, 19 patients received four IPL sessions with an acne filter in the right eye and a 590-nm filter in the left eye, and in group II, 14 patients received four IPL sessions with a 590-nm filter in the right eye and an acne filter in the left eye (Fig. 1). Two patients in group I and one in group II were lost to follow up. Measurements of the remaining 60 eyes of 30 patients were used for statistical analysis. During the IPL treatment, patients were allowed to use 0.15% sodium hyaluronate (New Hyaluni, Taejoon, Seoul, South Korea) and no other treatment, including warm compresses and lid scrubs.

## Data Availability

The datasets generated during and/or analyzed during the current study are available from the corresponding author on reasonable request.
